# Case report: POEMS syndrome masquerades as diabetic foot

**DOI:** 10.3389/fneur.2023.1336382

**Published:** 2024-01-26

**Authors:** Guang-Xin Zhou, Li Xiao, Yong-Min Bi, Fen Yang, Cai-Zhe Yang, Da Zhang

**Affiliations:** ^1^Department of Endocrinology, Air Force Medical Center, Air Force Medical University, Beijing, China; ^2^Department of Nuclear Medicine, Air Force Medical Center, Air Force Medical University, Beijing, China; ^3^Department of Neurology, Air Force Medical Center, Air Force Medical University, Beijing, China

**Keywords:** diabetic foot, POEMS syndrome, peripheral neuropathy, endocrinological manifestations, muscle weakness

## Abstract

We present the case of a 54-year-old woman with reasonable blood sugar control who presented with a diabetic foot combined with severe peripheral neuropathy and vascular disease. Lower limb muscle weakness, muscle atrophy, skin pigmentation, and emaciation were also observed. Although her muscle strength improved after glucocorticoid treatment, it remained challenging to account for the other symptoms in this particular patient with chronic inflammatory demyelinating polyneuropathy. Plump liver and spleen, hidden bone lesions combined with seemingly unexplained cerebral infarction, and serous effusion led us to suspect polyneuropathy, organomegaly, endocrinopathy, monoclonal gammopathy, and skin changes (POEMS) syndrome. The abnormal proliferation of monoclonal plasma cells and a significant increase in vascular endothelial growth factor (VEGF) levels confirmed the diagnosis of POEMS syndrome. After 1 month of treatment with lenalidomide and dexamethasone, the diabetic foot ulcers healed, and the symptoms of myasthenia and fatigue improved. Diabetic feet may represent only the tip of the iceberg of an underlying POEMS syndrome. Our report aimed to increase awareness of this rare yet significant situation, advocating for the prompt identification and treatment of POEMS syndrome.

## Introduction

A diabetic foot is a chronic complication of diabetes. Approximately 19–34% of patients with diabetes are likely to suffer from diabetic foot disease in their lifetime (1). Foot ulcers are a major cause of hospital admission for diabetes, and patients with foot ulcers have a 10-fold higher risk of amputation than those without foot ulcers (2). Prompt treatment and care of patients with diabetes with foot ulcers are essential in reducing the risk of amputation and avoiding other adverse consequences.

Studies have shown that the incidence of diabetic foot disease is closely associated with diabetic peripheral neuropathy (3, 4). However, peripheral neuropathy in patients with diabetes is not caused by hyperglycemia. Pierre et al. reviewed the clinical and pathological data of 100 consecutive patients with diabetes who had symptomatic neuropathy; one-third of whom had neuropathy unrelated to diabetes. Non-diabetic neuropathy includes conditions such as chronic inflammatory demyelinating polyneuropathy (CIDP) (9%), alcoholism (5%), vasculitis (4%), and monoclonal gammopathy (3%) (5). Monoclonal antibody peripheral neuropathy is a rare but important cause of neuropathy and often indicates a serious underlying disease, such as monoclonal gammopathy of undetermined significance (MGUS), multiple myeloma, amyloidosis, Waldenström’s macroglobulinemia, and polyneuropathy, organomegaly, endocrinopathy, monoclonal gammopathy, and skin changes (POEMS) syndrome (6).

POEMS syndrome is a rare plasma cell disease characterized by polyneuropathy, organomegaly, endocrinopathy, monoclonal proteins, and skin changes. The predilection age for POEMS syndrome is 40–60 years (7, 8), and the prevalence rate is 0.3/100,000 (8). The neurological symptoms, endocrine manifestations, and anemia caused by POEMS syndrome gradually worsen; therefore, early diagnosis and intervention are crucial (9). However, the rates of missed diagnoses and misdiagnoses are high because of the rarity of POEMS syndrome, multisystem involvement, and high clinical heterogeneity. The most common initial symptoms are peripheral neuropathy and increased water load, such as edema and serous fluid (10). The first department that a patient with POEMS syndrome visits is typically the department of neurology, nephrology, or gastroenterology. However, it has not been reported whether patients presenting with diabetic feet are eventually diagnosed with POEMS syndrome. This study reports a case of POEMS syndrome with diabetic foot as the chief complaint. It reviews the relevant literature to improve the diagnosis and treatment of POEMS syndrome by primary care physicians.

## Case presentation

**Figure 1:**
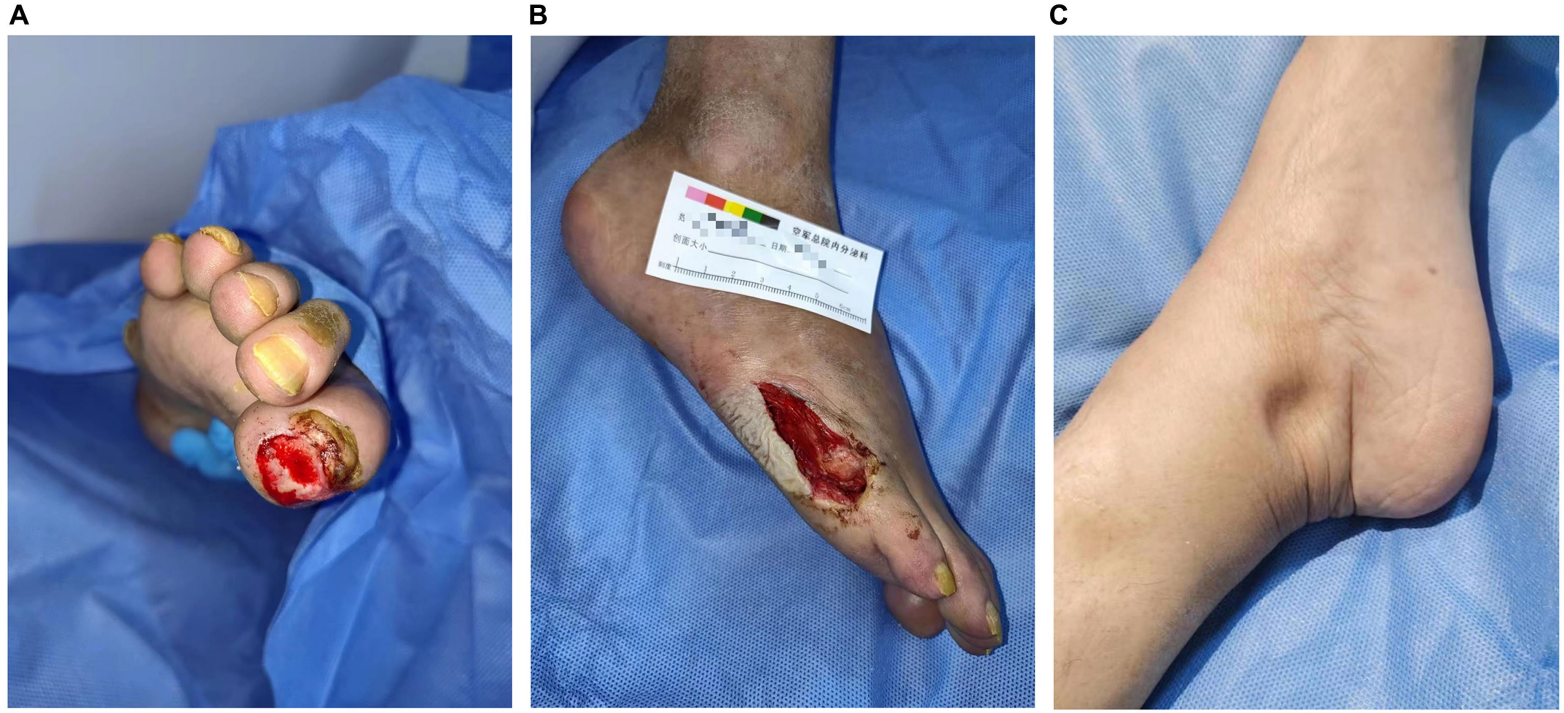
Clinical images of the patient. Ulcers on the first toe **(A)** and anterolateral foot **(B)** of the right foot are refractory. Sunken edema is evident in the medial malleolus **(C)**.

A 54-year-old woman with 8 years of diabetes was admitted to our center on 13 September 2022. Her chief complaint included right foot ulcerations that had occurred 8 months before her visit. Cyanosis due to arterial occlusion was observed in the lower extremities. The first and fifth toes developed refractory ulcerations upon bone exposure (Figure 1). Interventional therapy was performed twice to improve blood supply to the right foot with only modest efficacy. The patient had a poor appetite, muscular weakness, and fatigue, with a weight loss of approximately 10 kg within 2 years. She was allergic to penicillin and had a 10-year history of hypertension, stable blood pressure control, and a 2-year history of cataracts. A right oophorectomy was performed for a benign tumor in the right ovary. Physical examination revealed a slightly poor mental state, significant emaciation (BMI 16.85 kg/m^2^), skin pigmentation, and thickened hair on the lips and legs. The thenar and interphalangeal muscles of both hands were atrophic, with upper and lower limb muscle strengths at level 4. The pulsation of the bilateral femoral and popliteal arteries was normal, whereas the pulsation of the bilateral dorsalis pedis and posterior tibial arteries was weakened. She experienced weakened sensations of pressure, pain, warmth, and vibration in both feet, and the ankle reflex was absent. Mild concave edema was observed in both the lower limbs (Figure 1).

**Figure 2:**
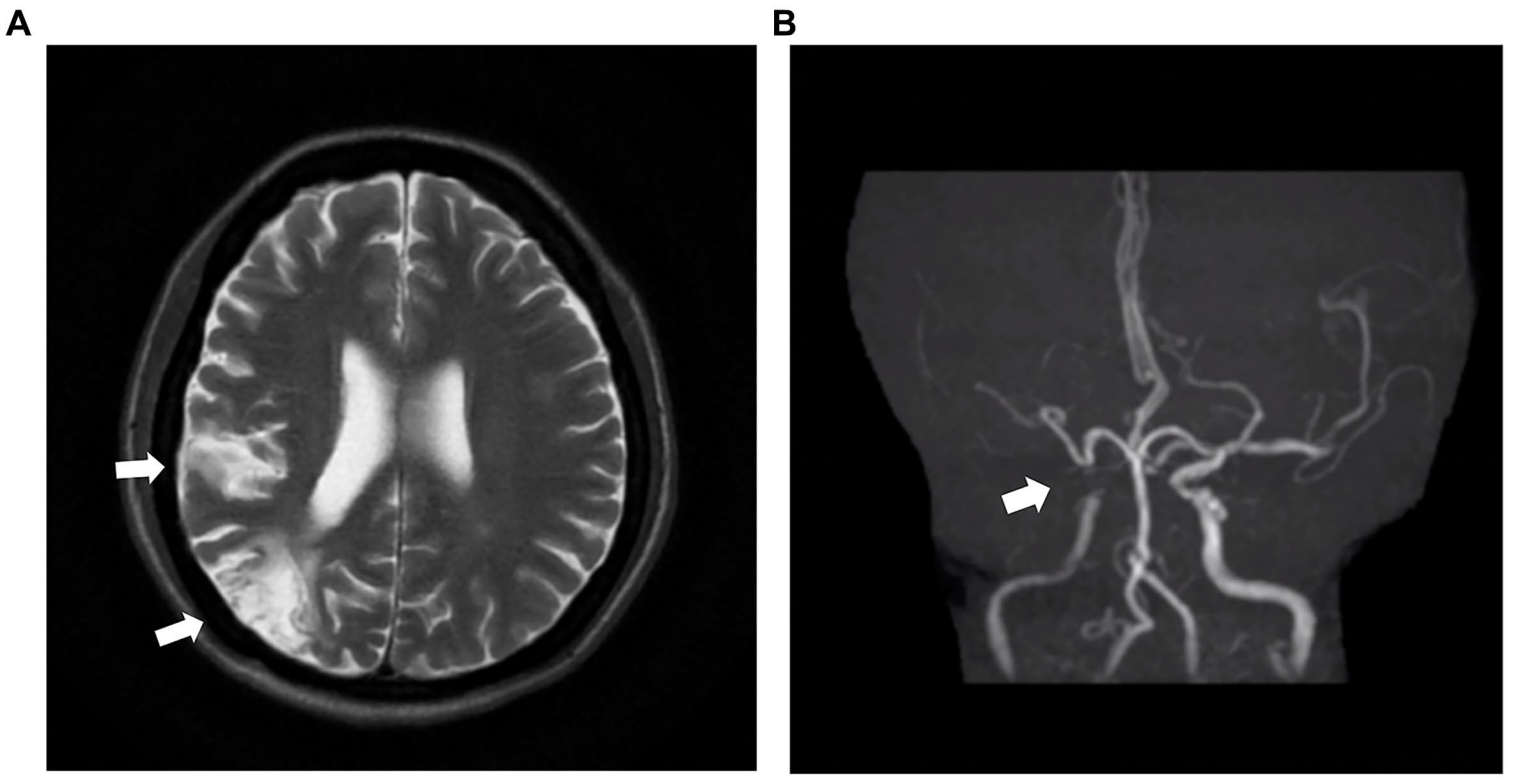
MRI and MRA images of the head of the patient. **(A)** The head MRI shows two infarctions at the right parietal lobe and temporo-occipital junction (white arrow). **(B)** The right internal carotid artery and right middle cerebral artery demonstrate severe stenosis and are almost occluded (white arrow). MRI: magnetic resonance imaging. MRA: magnetic resonance angiography.

**Figure 3:**
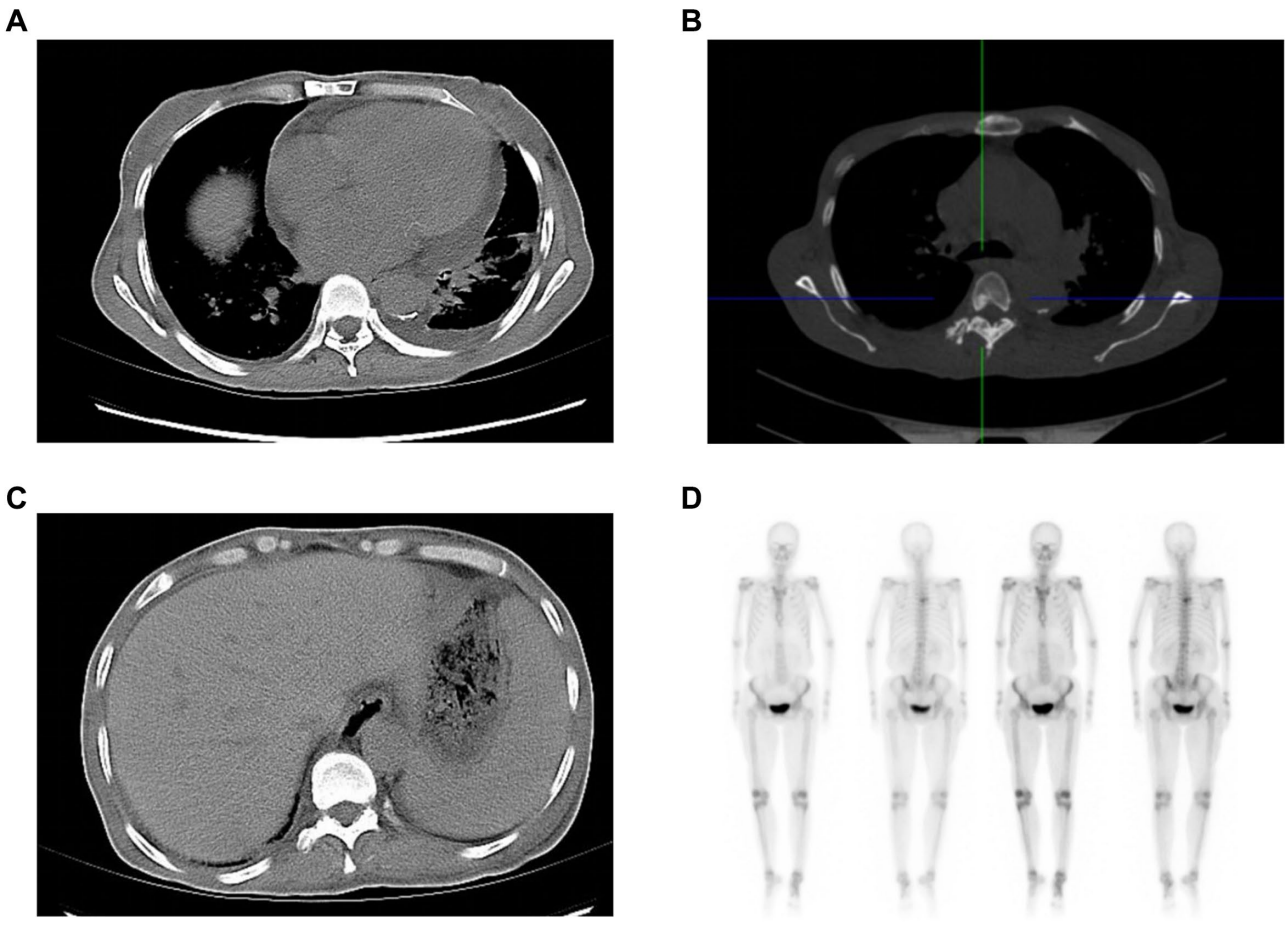
Clues from the chest and abdominal CT. The soft-tissue window of chest CT shows an enlarged heart, and pericardial and pleural effusion **(A)**, while the bone window of chest CT reveals osteosclerotic changes of vertebral body **(B)**. Abdominal CT indicates hepatosplenomegaly **(C)**. The radionuclide scan demonstrates a slight concentration of radioactivity on the fifth thoracic vertebral body **(D)**. CT: computed tomography.

Laboratory examination showed satisfactory blood glucose control, normal hemoglobin, and a lack of elevation of inflammatory markers, such as erythrocyte sedimentation rate and C-reactive protein; however, elevated platelet levels (593 × 10^9^/L) were noted. Serum calcium (2.14 mmol/L), serum phosphorus (1.50 mmol/L), and uric acid levels (357 μmol/L) were normal. The liver, kidney function, and thyroid function were normal, while the levels of estradiol (E2 114.73 pmol/L), luteinizing hormone (LH 0.98 mIU/mL; reference value, 10.87–58.64 mIU/mL), and follicle-stimulating hormone (FSH 1.46 mIU/mL; reference value, 16.74–113.59 mIU/mL) were low. Serum cortisol was slightly raised (8 am 511.70 nmol/L; 0 am 234.50 nmol/L) along with elevated adrenocorticotropic hormone (ACTH 124.30 pg/mL; reference value, 6.0–48.0 pg/mL). Immunoglobulin levels were normal. Magnetic resonance imaging of the head revealed two infarctions in the right parietal lobe and temporo-occipital junction due to the occlusion of the right internal carotid artery and right middle cerebral artery (Figure 2). Following a detailed inquiry into the medical history, we discovered that transient coughing occurred upon drinking water. Incontinence was present without hemiplegia, and the patient was inattentive. Neuroelectrophysiological examination showed that the conduction velocity of the motor and sensory nerves of the limbs was reduced, the latency of the F wave of the upper limbs was prolonged, and the F wave of the left lower limbs was not elicited, suggesting severe involvement of multiple peripheral sensory and motor nerves with axonal and demyelinating damage in the limbs. Neuropathy was more severe in the lower limbs than in the upper limbs. Subsequently, a lumbar puncture examination was performed. There was no evidence of protein–cell separation in the cerebrospinal fluid. However, considering the patient’s medical history, symptoms, signs, and neuroelectrophysiological examination results, the possibility of CIDP was suspected. Prednisone was administered with the permission of the patient and her husband. After 1 month of medication, the patient’s symptoms of limb weakness improved, and the upper and lower limb muscle strengths returned to approximately level 5. However, there was no improvement in ankle edema. Imaging findings, including a small amount of pleural and pericardial effusion, mild pulmonary hypertension, plump liver and spleen, and vertebral osteosclerosis (Figure 3), provided clues for investigating the underlying disease. Blood light chain λ was 987.00 mg/dL (reference value, 280–665 mg/dL). An abnormal band, accounting for approximately 7.0%, was observed in the γ region during serum protein electrophoresis. Type IgG-λ M protein was determined by serum immunofixation electrophoresis. Bone marrow puncture and biopsy revealed active proliferation of the bone marrow, a normal proportion of plasma cells, and a few scattered plasma cells in the bone marrow. Immunohistochemical analysis revealed the absence of significant monoclonal cells. Additionally, the level of vascular endothelial growth factor (VEGF) was substantially elevated, with a value of 1145.72 pg/mL (normal range 0–142 pg/mL).

### Diagnostic assessment

The latest diagnostic criteria for POEMS syndrome were updated by Dispenzieri (11) in 2019, which included the following parameters: ① necessary conditions: multiple peripheral neuropathies and monoclonal plasma cell proliferation. ② Primary conditions: sclerotic bone lesions, Castleman’s disease, and elevated serum VEGF. ③ Secondary conditions: organ enlargement (splenomegaly, hepatomegaly, and lymphadenopathy), extravascular volume overload (peripheral edema, pleural effusion, or ascites), endocrine abnormalities (pituitary, thyroid, parathyroid, adrenal, gonad, and pancreas), skin changes (pigmentation, hirsutism, hemangioma, Raynaud’s phenomenon, pale nail, and polyemia), papilledema, and thrombocytosis or polycythemia. The diagnosis of POEMS syndrome can be made only when at least one primary and one secondary condition exist.

The patient met two necessary diagnostic criteria: two of the main diagnostic criteria, including sclerotic bone lesions and elevated serum VEGF, and five secondary diagnostic criteria, including organ enlargement, extravascular volume overload, endocrine abnormalities, skin changes, and thrombocytosis. She was diagnosed with POEMS syndrome and type 2 diabetes mellitus with a diabetic foot.

### Treatment, outcome, and follow-up

We thoroughly explained to the patient and her husband the benefits and limitations of different treatment methods, including autologous hematopoietic stem cell transplantation (ASCT), melphalan plus dexamethasone (MDex), and lenalidomide plus dexamethasone (LDex) (Table 1). Finally, the patient underwent LDex treatment. She received lenalidomide 25 mg once daily for 3 weeks in a 4-week cycle, followed by a 1-week break, along with dexamethasone 40 mg once a week. Following 1 month of treatment with LDex, the foot ulcer was healed. Throughout the follow-up period until July 2023, the blood glucose level was maintained between 5 and 6 mmol/L, and the patient took LDex consistently. No adverse events or other discomforts were reported. Muscle weakness and fatigue symptoms significantly improved, and the patient could walk 600–700 m independently. The patient’s weight in July 2023 was 51.5 kg, which was comparably higher than the weight of 39 kg at the time of discharge.

**Table 1:** Advantages and disadvantages of first-line treatments for POEMS syndrome.

Treatment	ASTC	MDex	LDex
Benefits	Highest CRH, CRV, and PFS especially in high-risk patients	CRH, CRV similar to ASCT in low-risk patients	Response rates and OS similar to ASCT, quicker response
Limitations	Pre-engraftment syndrome, secondary tumor	Treatment-related stem cell damage, secondary myelodysplastic syndrome, and leukemia in myeloma patients	Cytopenia, other malignancies risks

ASTC, autologous hematopoietic stem cell transplantation; MDex, melphalan plus dexamethasone; LDex, lenalidomide plus dexamethasone; CRH, overall hematologic complete remission; CRV, vascular endothelial growth factor complete remission; OS, overall survival; PFS, progression-free survival.

## Discussion

A diabetic patient with reasonable blood sugar control was admitted to our hospital with a diabetic foot. Lower limb muscle weakness, muscle atrophy, skin pigmentation, and emaciation were observed in the patient. Although her muscle strength improved after glucocorticoid treatment, it was impossible to explain the other symptoms in the patient with CIDP. Plump liver and spleen, hidden bone lesions combined with seemingly unexplained cerebral infarction, and serous effusion led us to suspect POEMS syndrome.

The common misdiagnoses of POEMS syndrome include CIDP, MGUS, and multiple myeloma (12). Diagnosis can be made approximately 1–2 years after initial symptom presentation (13). A literature-based characteristics study of 1,946 cases of POEMS syndrome (10) showed that the most common initial symptoms were peripheral neuropathy (796) (60.44%), followed by extravascular volume overload (207) (15.72%), endocrine abnormalities (130) (9.87%), skin changes (106) (8.05%), and organomegaly (28) (2.13%). The initial symptoms of some patients were uncommon, including weight loss, diarrhea, nephritis, proteinuria, and pain, which accounted for 4% of the cases. Most patients present with other symptoms or signs at their first visit, and a range of symptoms are often overlooked as minor comorbidities (14). The patient in our study was admitted to our hospital with diabetic foot disease. It can be presumed that the diabetic foot may have been an opportunity for the patient to reach out to the doctor to seek medical help, as she was symptomatic for several days before the manifestation of the diabetic foot. Fortunately, during the diabetic foot workup, we found evidence of POEMS syndrome.

The symptoms primarily manifested as severe nervous system involvement. For patients with diabetes, long-term hyperglycemia not only has a direct killing effect on nerve cells but also causes peripheral microvascular disease, affects the nutritional supply of nerve cells, leading to ischemia and hypoxia of nerve cells, and finally leads to the development of diabetic peripheral neuropathy (DPN). DPN is primarily a sensory nerve disorder, while the motor nerve injury is mild. The neuroelectrophysiology of DPN is mainly characterized by axonal damage and secondary demyelination, which differ from CIDP (15). The clinical manifestations of POEMS syndrome are similar to those of CIDP and are characterized by subacute or chronic symmetric demyelinating polyradiculopathy (16). Although the electrophysiological results of patients with POEMS syndrome indicate that they are more likely to have a rare conduction block, more neurogenic injury in the muscles of the lower limbs than in the upper limbs, and rare skin sympathetic response abnormalities (17, 18), there are no studies to propose specific electrophysiological diagnostic criteria for CIDP and POEMS syndrome, which poses certain challenges to our diagnosis in the early stages. Niu J demonstrated that patients with CIDP and POEMS syndrome have different ultrasonic features. The nerve cross-sectional area enlargement was more homogeneous along the same nerve in patients with POEMS syndrome. Applying neuro-ultrasound in the early stages could significantly improve the differential diagnosis between the two diseases (19).

There are other clues for an early diagnosis. Endocrine diseases are an important feature of POEMS syndrome. Endocrine disorders in patients with POEMS syndrome mainly manifest as hypogonadism, hypothyroidism, adrenal insufficiency, and diabetes (20, 21). The patient’s sex hormone levels suggested hypogonadotropic hypogonadism, which was ignored before the diagnosis of POEMS syndrome. Although ACTH increased, cortisol levels were at the normal high limit, urinary free cortisol was elevated, and the patient had no typical Cushing’s appearance, such as a full moon face, buffalo back, or purple striae. No space-occupying lesions were observed in the pituitary gland, lungs, abdomen, or pelvic cavity. The patient’s adrenal hormone abnormality was considered to be pseudo-Cushing’s syndrome, caused by stress. Some patients with POEMS syndrome may also have multiple endocrine dysfunctions. Although POEMS syndrome with endocrine abnormalities as the first manifestation is rare, endocrinologists should be aware of the possibility of POEMS syndrome in patients with multiple endocrine gland dysfunctions.

The patient’s blood glucose and blood pressure were under control, with no history of smoking, drinking, or genetic disease. However, at the age of 54 years, the patient exhibited serious occlusion of the cerebral and lower extremity arteries. VEGF increases vascular permeability and promotes vascular endothelial cell proliferation and neovascularization, leading to systemic vascular stenosis/occlusion (22). A study by Sugiyama demonstrated that approximately half of patients with POEMS syndrome exhibited major cerebral artery stenosis/occlusion on initial MRA/CTA (23). Multiple vascular involvement was observed in approximately one-third of all patients with POEMS syndrome. Severe peripheral vascular and nervous system diseases can result in the development of refractory foot ulcers.

An analysis of 107 cases of POEMS syndrome by Miest (24) showed that 90% of patients had skin manifestations, among which skin pigmentation and hemangioma were the most common (47%), followed by hirsutism (38%). Vascular skin changes were observed in 62% of patients, such as cyanosis (34%), Raynaud’s phenomenon (20%), congestion/erythema (20%), facial flushing (16%), and skin redness (11%). VEGF stimulates melanogenesis, which leads to skin pigmentation (11). VEGF can also act directly on dermal papillary cells or stimulate the local vascular system, causing hirsutism (25). Elevated VEGF levels are associated with polyneuropathy, organ enlargement, endocrine disease, polyproteins, and skin changes in POEMS syndrome (26), and their concentration is closely related to disease progression, treatment effects, and survival rate (27, 28). The skin changes in this patient were closely related to the significant increase in VEGF levels, which served as a potential clue for the diagnosis of POEMS syndrome.

There is currently no standard therapy for POEMS syndrome (29, 30). The treatment principle was based on the degree of plasma cell infiltration. The patient had multiple skeletal lesions, including diffuse bone marrow involvement, and was treated with systemic therapy (11). ASCT, MDex, and LDex have shown good therapeutic effects in systemic treatment; however, the choice of the best treatment option remains unclear (31). At Peking Union Medical College Hospital (32), MDex was the most frequently used first-line therapy (50.6%) but was replaced by ASCT in 2011–2014 (47.5%) and then LDex in 2015–2019 (37.6%). Studies have shown that LDex can induce rapid neurological and hematologic responses (33) and result in a similar overall survival rate increase as ASCT and MDex (31). It was reported that 75–95% of patients demonstrated significant improvement in clinical and VEGF symptoms upon LDex treatment (11), making it the preferred choice of treatment. However, the optimal duration of LDex remains controversial. Although prolonged treatment may have a positive effect on achieving more progression-free survival (PFS), it is necessary to consider the side effects of treatment, such as cytopenia, and the risk of other malignancies (34).

The common causes of death in patients with POEMS syndrome include respiratory and circulatory failure, progressive malnutrition, infection, and renal failure. A study by Taxiarchis et al. (35) found that younger age, albumin greater than 3.2 g/dL at diagnosis, and a complete hematologic response were associated with good survival rates. The 10-year survival rates of patients diagnosed before and after 2003 were 55 and 79%, respectively (11). The prognosis of patients with POEMS syndrome is better than the previously reported values, with a median survival duration of 165 months regardless of the number of syndrome features, bone lesions, or plasma cells observed at diagnosis (7). The 6-year PFS rate was greater than 50% in patients receiving modern therapy and 88% in patients with a hematologic complete response (11). In this particular case, it took less than 10 months from the onset of the first symptoms of peripheral neuropathy to reach a conclusive diagnosis. Our diagnosis was comparatively prompt, and the patient demonstrated a satisfactory outcome after LDex treatment.

## Conclusion

Our report indicates that POEMS syndrome is easily missed and misdiagnosed in the early stages, not only because of its rarity but also because the symptoms are complex and can mimic those of other disorders. Diabetic feet are the tip of the iceberg in POEMS syndrome. Patients with POEMS syndrome may seek medical assistance for diverse initial symptoms. Primary care physicians should be aware of this issue, particularly in patients with multiple systemic symptoms.

## Data availability statement

The original contributions presented in the study are included in the article/supplementary material, further inquiries can be directed to the corresponding author.

## Ethics statement

The studies involving humans were approved by Ethics Committee of Air force Medical Center. The studies were conducted in accordance with the local legislation and institutional requirements. The participants provided their written informed consent to participate in this study. Written informed consent was obtained from the individual(s) for the publication of any potentially identifiable images or data included in this article.

## Author contributions

G-XZ: Writing – original draft. LX: Writing – original draft. Y-MB: Writing – review & editing. FY: Writing – review & editing. C-ZY: Writing – review & editing. DZ: Writing – review & editing.
